# Supplementary material for: Secreted spermidine synthase reveals a paracrine role for PGC1α-induced growth suppression in prostate cancer
Source: Cell Death Dis. 2025 Apr 23;16(1):330. doi: 10.1038/s41419-025-07639-4 (PMC12019391; doi:10.1038/s41419-025-07639-4)
Supplement: Supplementary file 1 — Supplementary Figure 1 [file 41419_2025_7639_MOESM1_ESM.pptx]

## Slide 1
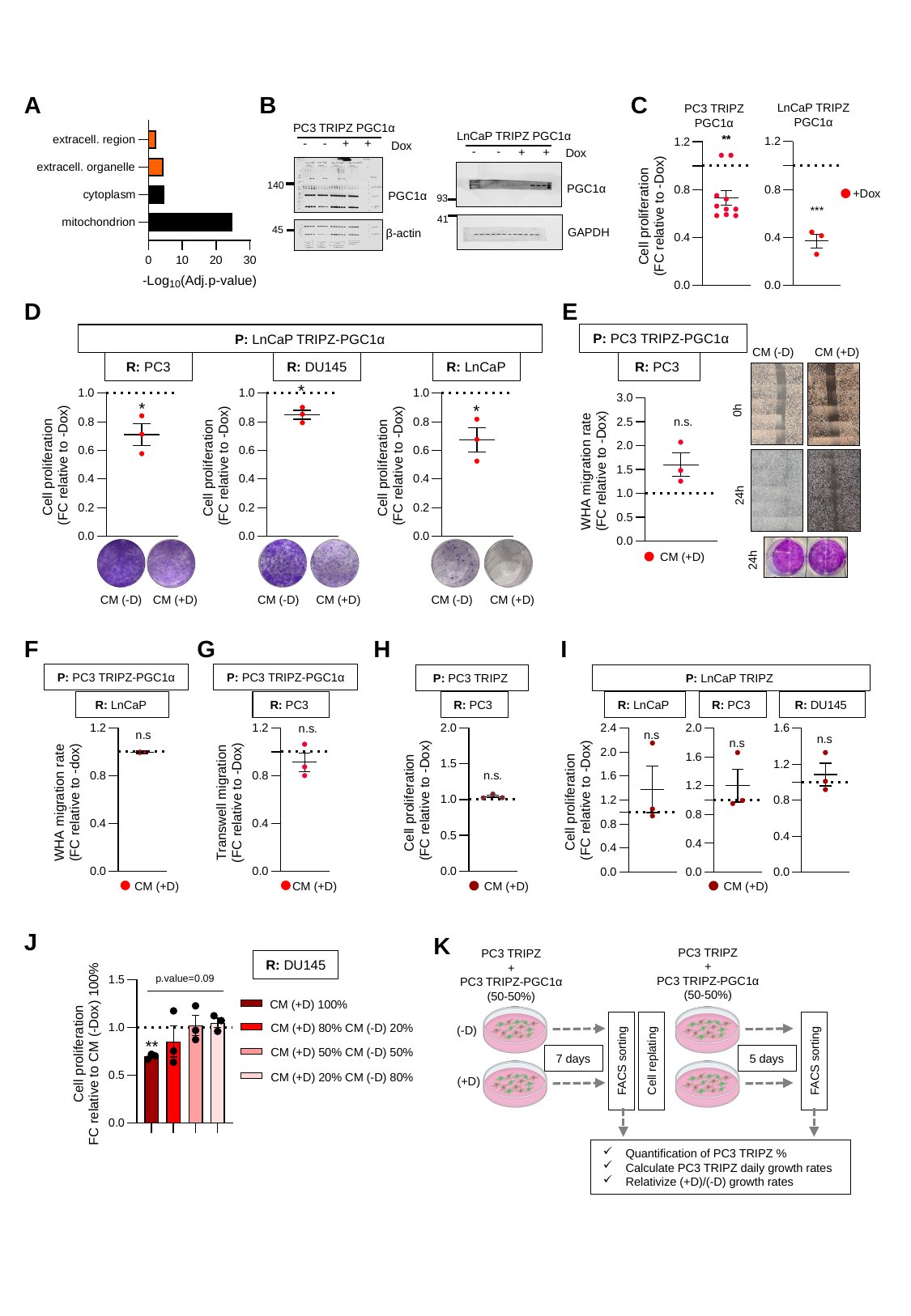

A
B
C
LnCaP TRIPZ PGC1α
PC3 TRIPZ PGC1α
PC3 TRIPZ PGC1α
+
-
-
+
Dox
PGC1α
β-actin
LnCaP TRIPZ PGC1α
-
-
+
+
Dox
140
PGC1α
+Dox
93
41
45
GAPDH
D
E
P: PC3 TRIPZ-PGC1α
P: LnCaP TRIPZ-PGC1α
CM (-D)
CM (+D)
R: PC3
R: PC3
R: DU145
R: LnCaP
*
*
*
0h
24h
CM (+D)
24h
CM (-D)
CM (+D)
CM (-D)
CM (+D)
CM (-D)
CM (+D)
F
G
H
I
P: LnCaP TRIPZ
P: PC3 TRIPZ-PGC1α
P: PC3 TRIPZ-PGC1α
P: PC3 TRIPZ
R: LnCaP
R: PC3
R: PC3
R: LnCaP
R: PC3
R: DU145
n.s
n.s
n.s
CM (+D)
CM (+D)
CM (+D)
CM (+D)
J
K
PC3 TRIPZ
 +
PC3 TRIPZ-PGC1α
(50-50%)
PC3 TRIPZ
 +
PC3 TRIPZ-PGC1α
(50-50%)
R: DU145
CM (+D) 100%
CM (+D) 80% CM (-D) 20%
(-D)
CM (+D) 50% CM (-D) 50%
7 days
5 days
FACS sorting
Cell replating
FACS sorting
CM (+D) 20% CM (-D) 80%
(+D)
Quantification of PC3 TRIPZ %
Calculate PC3 TRIPZ daily growth rates
Relativize (+D)/(-D) growth rates
