# Supplementary material for: Secreted spermidine synthase reveals a paracrine role for PGC1α-induced growth suppression in prostate cancer
Source: Cell Death Dis. 2025 Apr 23;16(1):330. doi: 10.1038/s41419-025-07639-4 (PMC12019391; doi:10.1038/s41419-025-07639-4)
Supplement: Supplementary file 2 — Supplementary Figure 2 [file 41419_2025_7639_MOESM2_ESM.pptx]

## Slide 1
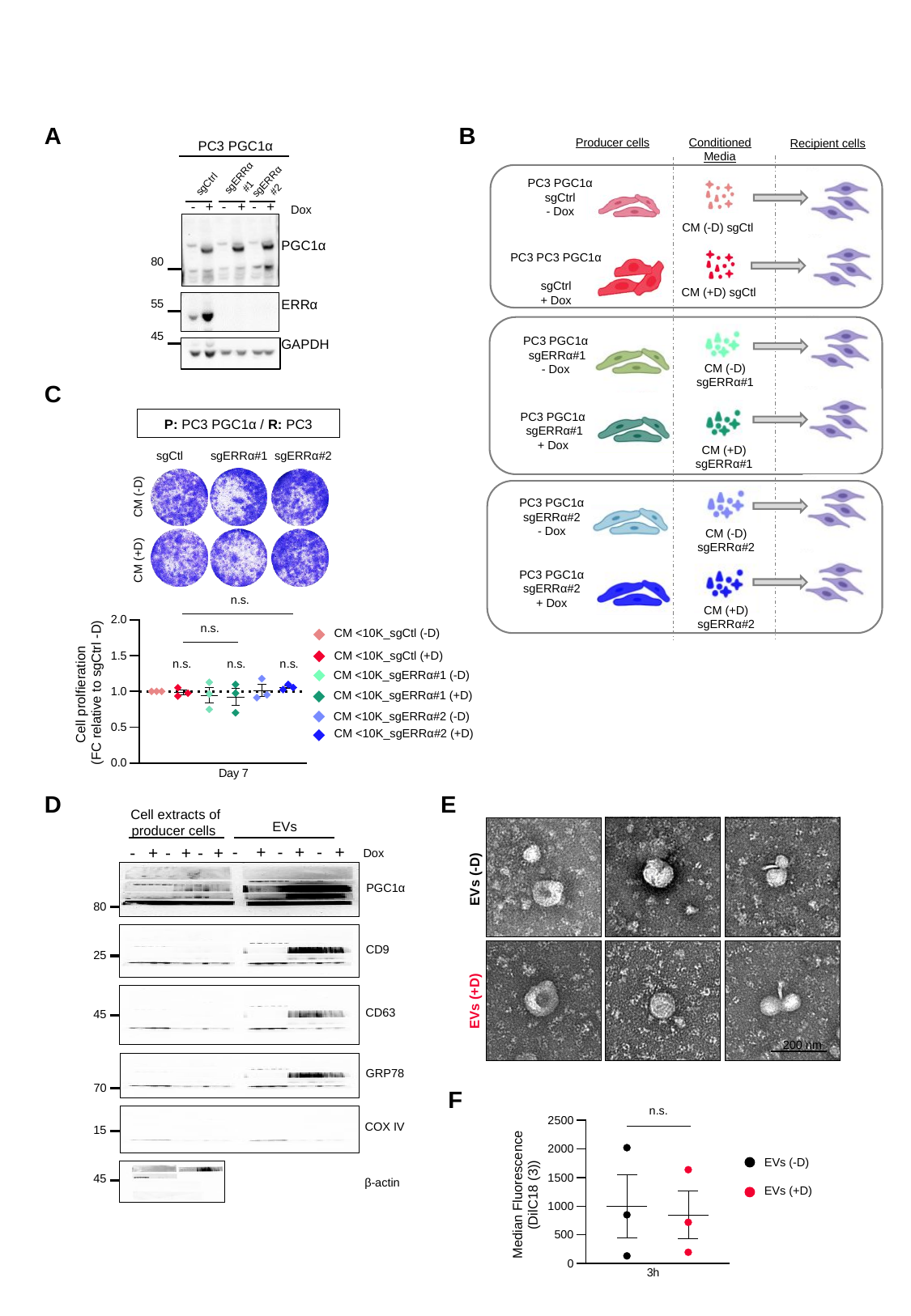

B
A
Producer cells
Conditioned Media
PC3 PGC1α
Recipient cells
sgERRα
#1
sgERRα
#2
sgCtrl
PC3 PGC1α
sgCtrl
- Dox
-
+
-
+
-
+
Dox
CM (-D) sgCtl
PGC1α
80
PC3 PC3 PGC1α
sgCtrl
+ Dox
CM (+D) sgCtl
ERRα
55
45
GAPDH
PC3 PGC1α
 sgERRα#1
- Dox
CM (-D) sgERRα#1
C
P: PC3 PGC1α / R: PC3
PC3 PGC1α
 sgERRα#1
+ Dox
CM (+D) sgERRα#1
sgCtl
sgERRα#1
sgERRα#2
CM (-D)
PC3 PGC1α
sgERRα#2
- Dox
CM (-D) sgERRα#2
CM (+D)
PC3 PGC1α
sgERRα#2
+ Dox
CM (+D) sgERRα#2
CM <10K_sgCtl (-D)
CM <10K_sgCtl (+D)
CM <10K_sgERRα#1 (-D)
CM <10K_sgERRα#1 (+D)
CM <10K_sgERRα#2 (-D)
CM <10K_sgERRα#2 (+D)
D
E
 Cell extracts of
producer cells
 EVs
-
+
-
+
-
+
-
+
-
+
-
+
Dox
EVs (-D)
PGC1α
80
CD9
25
EVs (+D)
CD63
45
200 nm
GRP78
70
F
COX IV
15
EVs (-D)
45
β-actin
EVs (+D)
