# Supplementary material for: Secreted spermidine synthase reveals a paracrine role for PGC1α-induced growth suppression in prostate cancer
Source: Cell Death Dis. 2025 Apr 23;16(1):330. doi: 10.1038/s41419-025-07639-4 (PMC12019391; doi:10.1038/s41419-025-07639-4)
Supplement: Supplementary file 3 — Supplementary Figure 3 [file 41419_2025_7639_MOESM3_ESM.pptx]

## Slide 1
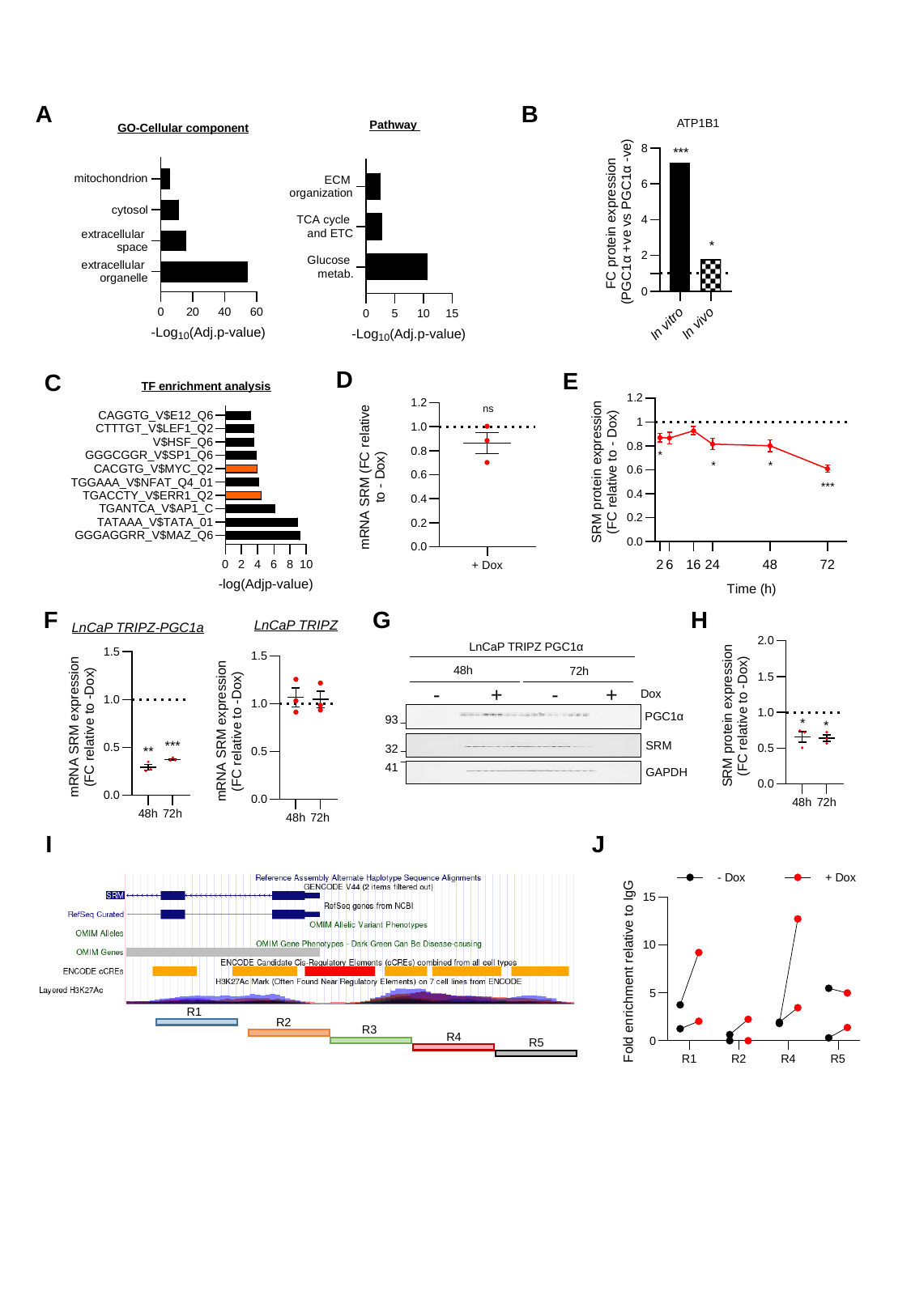

A
B
ATP1B1
Pathway
GO-Cellular component
***
*
D
E
C
TF enrichment analysis
F
G
H
LnCaP TRIPZ
LnCaP TRIPZ-PGC1a
LnCaP TRIPZ PGC1α
48h
72h
+
-
+
-
Dox
PGC1α
_
93
SRM
_
32
_
41
GAPDH
I
J
R1
R2
R3
R4
R5
