# Supplementary material for: Secreted spermidine synthase reveals a paracrine role for PGC1α-induced growth suppression in prostate cancer
Source: Cell Death Dis. 2025 Apr 23;16(1):330. doi: 10.1038/s41419-025-07639-4 (PMC12019391; doi:10.1038/s41419-025-07639-4)
Supplement: Supplementary file 4 — Supplementary Figure 4 [file 41419_2025_7639_MOESM4_ESM.pptx]

## Slide 1
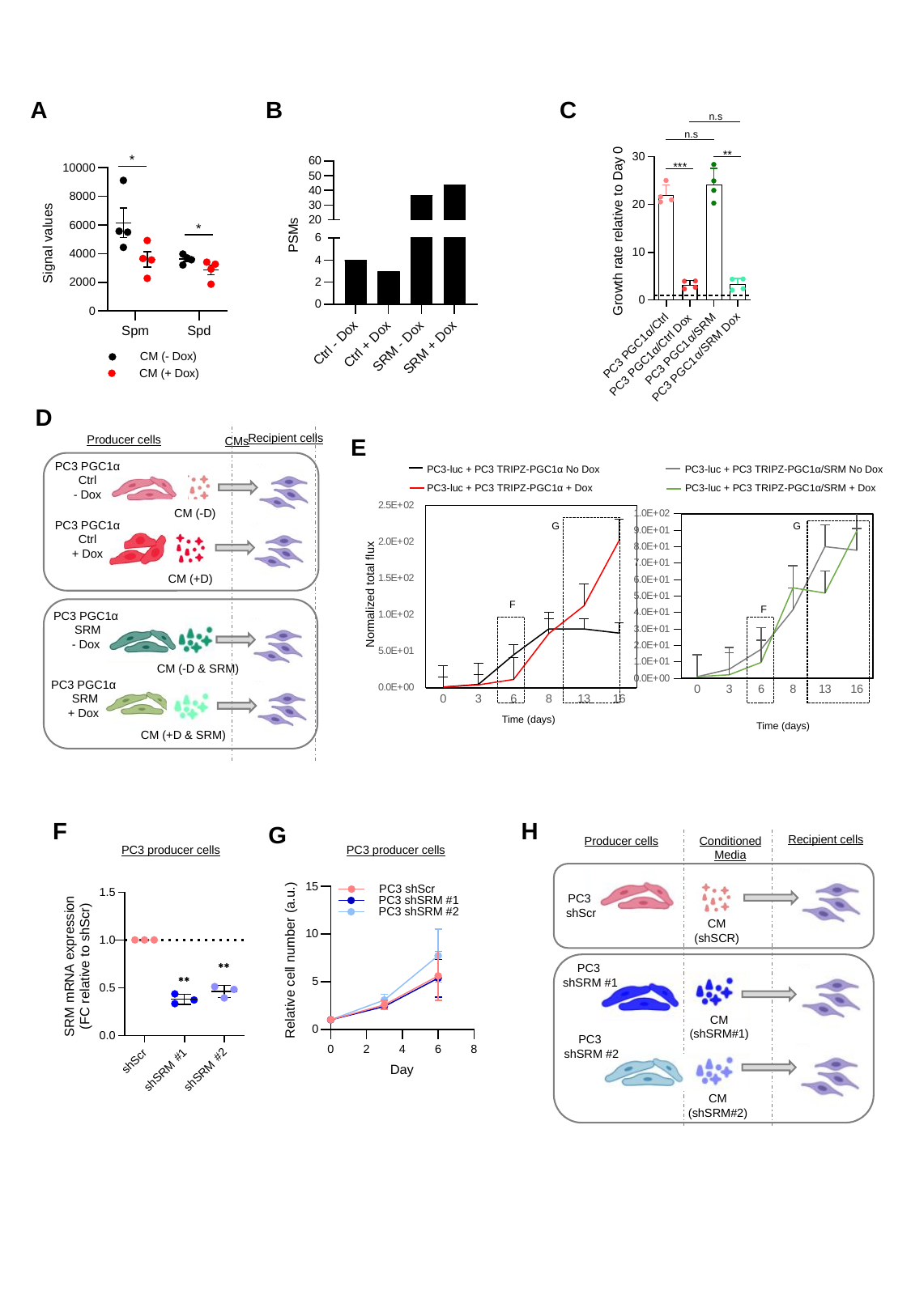

A
B
C
*
*
D
E
Recipient cells
Producer cells
CMs
PC3-luc + PC3 TRIPZ-PGC1α No Dox
PC3-luc + PC3 TRIPZ-PGC1α/SRM No Dox
PC3 PGC1α
Ctrl
- Dox
PC3-luc + PC3 TRIPZ-PGC1α + Dox
PC3-luc + PC3 TRIPZ-PGC1α/SRM + Dox
### Chart
| Category | PC3 PGC1A SRM No Dox | PC3 PGC1A SRM Dox |
|---|---|---|
| 0 | 1.0 | 1.0 |
| 3 | 5.529237840543866 | 2.1675873967663737 |
| 6 | 17.69015053837344 | 9.69307762051653 |
| 8 | 41.74426562657406 | 55.04802636563876 |
| 13 | 80.09358490973842 | 51.88185128228723 |
| 16 | 77.89444266911227 | 89.79850044102669 |
### Chart
| Category | PC3 PGC1A Clover Ctrl No Dox | PC3 PGC1A Clover Ctrl Dox |
|---|---|---|
| 0 | 1.0 | 1.0 |
| 3 | 4.708618258995088 | 4.050686394740288 |
| 6 | 45.03941967674928 | 11.400907692119988 |
| 8 | 80.62988808327458 | 74.1852411787006 |
| 13 | 80.59883529241587 | 112.24420671021771 |
| 16 | 74.92085740687043 | 201.8016476574715 |CM (-D)
G
G
PC3 PGC1α
Ctrl
+ Dox
CM (+D)
Normalized total flux
F
F
PC3 PGC1α
 SRM
- Dox
CM (-D & SRM)
PC3 PGC1α
 SRM
+ Dox
Time (days)
Time (days)
CM (+D & SRM)
F
H
G
Producer cells
Conditioned Media
Recipient cells
PC3 producer cells
PC3 producer cells
PC3
shScr
CM (shSCR)
PC3
shSRM #1
CM (shSRM#1)
PC3
shSRM #2
CM (shSRM#2)
