# Supplementary material for: Secreted spermidine synthase reveals a paracrine role for PGC1α-induced growth suppression in prostate cancer
Source: Cell Death Dis. 2025 Apr 23;16(1):330. doi: 10.1038/s41419-025-07639-4 (PMC12019391; doi:10.1038/s41419-025-07639-4)
Supplement: Supplementary file 5 — Supplementary Figure 5 [file 41419_2025_7639_MOESM5_ESM.pptx]

## Slide 1
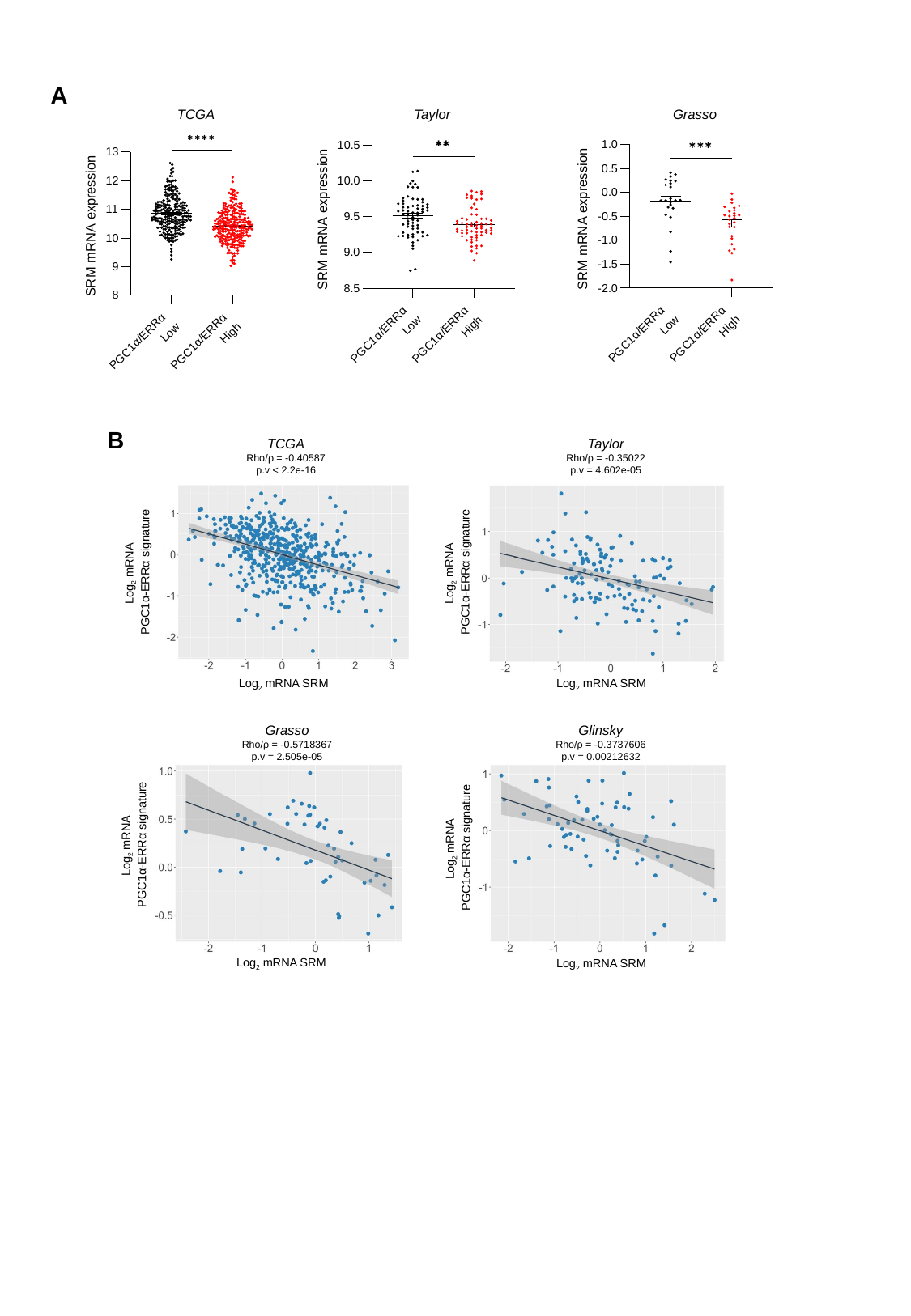

A
TCGA
Taylor
Grasso
B
TCGA
Rho/ρ = -0.40587
p.v < 2.2e-16
Taylor
Rho/ρ = -0.35022
p.v = 4.602e-05
Log2 mRNA
PGC1α-ERRα signature
Log2 mRNA
PGC1α-ERRα signature
Log2 mRNA SRM
Log2 mRNA SRM
Grasso
Rho/ρ = -0.5718367
p.v = 2.505e-05
Glinsky
Rho/ρ = -0.3737606
p.v = 0.00212632
Log2 mRNA
PGC1α-ERRα signature
Log2 mRNA
PGC1α-ERRα signature
Log2 mRNA SRM
Log2 mRNA SRM
