# Supplementary material for: Secreted spermidine synthase reveals a paracrine role for PGC1α-induced growth suppression in prostate cancer
Source: Cell Death Dis. 2025 Apr 23;16(1):330. doi: 10.1038/s41419-025-07639-4 (PMC12019391; doi:10.1038/s41419-025-07639-4)
Supplement: Supplementary file 6 — Supplementary figure legends [file 41419_2025_7639_MOESM6_ESM.pdf]

Supplementary Figure 1. A. Gene Ontology Cellular Component enrichment analysis of the genes differentially expressed upon PGC1α re-expression in PC3 cells (GSE75193). **B**. Western blot analysis of PGC1α re-expression in PC3 (left panel) and LnCaP (right panel) cells. **C**. Confirmation of the cell autonomous anti-proliferative effect of PGC1α re-expression in PC3 (left panel; n=10) and LnCaP (right panel; n=3) cells (already described in (1)). **D** Quantification of 2D- cell proliferation (crystal violet) of PC3, DU145 and LnCaP cells grown during 7 days with differential conditioned media produced by PGC1α non-expressing (CM (-D)) and expressing LnCaP cells (CM (+D)). **E-G**. Effect of conditioned media produced by PGC1α non-expressing and expressing PC3 cells on the migratory capacity of recipient PC3 and LnCaP cells (Wound healing-WHA assays: E-F and transwell migration assays: G; n=3). **H-I**. Quantification of 2D- cell proliferation (crystal violet) of AR positive and negative PCa cells grown with differential conditioned media produced by PC3 (H) and LnCaP (I)-TRIPZ control cells treated or not with doxycyline. **J**. Dose dependent effect of PGC1α-expressing PC3 cells´ conditioned media. Different percentages of PGC1a-expressing and non-expressing PC3 conditioned media were used to grow and monitor 2D proliferation of recipient DU145 cells (n=3). **K**. Summary of the experimental setting in the *in vitro* co-culture assays. In C to J panels, data are normalized to untreated doxycycline conditions, depicted by a black dotted line. P: producer cells. R: recipient cells. CM: conditioned media. D or Dox: doxycyline. n.s: not significant. FC: fold change. Statistics: one sample t-test with reference value 1 (one tailed C, D, J and two tailed E, F, G, H). * p.value < 0.05; ** p.value < 0.01; *** p.value < 0.001. Error bars indicate s.e.m.

Supplementary Figure 2. A. Western blot of PGC1α and ERRα on PC3 conditioned media producer cells. **B**. Schematic representation of the experimental approach to produced conditioned media from PGC1α non-expressing and expressing PC3 cells with or without deletion of ERRα. **C**. Quantification of 2D-cell proliferation (crystal violet) of PC3 (n=3) grown with the light fraction (<10kDa) of conditioned media produced by PGC1α non-expressing and expressing PC3 cells with or without deletion of ERRα. **D**. Protein expression analysis of different endomembrane markers (CD9, CD63 for extracellular vesicles, GRP78 for endoplasmic reticulum and COX IV for mitochondria) on isolated extracellular vesicles produced by PGC1α non-expressing and expressing PC3 cells. **E**. Electron microscopy characterization of EVs produced by doxycycline-induced and non-induced PC3 TRIPZ cells. Bar, 200 nm. **F**. FACS analysis of EVs uptake by PC3 cells after 3 hours of incubation with stained EVs produced by PGC1α non-expressing and expressing cells. R: recipient cells. CM: conditioned media. D or

Dox: doxycyline. n.s: not significant. Statistics: (B) one sample t-test with reference value 1 (between No Dox and Dox conditions) and paired-t-test (between Control Dox and sgERRα#1/sgERRα#2 Dox). * p.value < 0.05; ** p.value < 0.01; *** p.value < 0.001. Error bars indicate s.e.m.

Supplementary Figure 3. A. Functional enrichment analysis (left panel, GOCC-Cellular component; right panel, Pathway analysis) of genes encoding for the proteins differentially secreted by PGC1α expressing cells. **B.** Histogram showing the degree of change in the detection of ATP1B1 in *in vitro* conditioned media and *in vivo* interstitial liquid. **C**. Transcription factor enrichment analysis of genes encoding for the proteins differentially secreted by PC3 PGC1α-expressing cells. **D**. Quantification of SRM mRNA expression in PC3-TRIPZ_empty vector control cells treated or not with doxycycline (RT-qPCR, n=3). **E**. Quantification of SRM protein expression in PC3 PGC1α-expressing cells (WB, n=4) treated with doxycycline at different time points. **F**. Quantification of SRM mRNA expression in LnCaP PGC1α-expressing (left panel) and LnCaP-TRIPZ_empty vector (right panel) cells. Both cell types were treated with doxycycline for 48 and 72 hours. (RT-qPCR, n=3). **G-H**. Effect of PGC1α re-expression on SRM protein levels in LnCaP cells (representative WB, **G** and quantification, **H**, n=3) after 48 and 72 hours of doxycycline treatment. **I**. Schematic representation of the different genomic regions (R) located in SRM promoter and monitored in chromatin immunoprecipitation assays. **J**. Chromatin immunoprecipitation of exogenous Pgc1α in PC3-PGC1α expressing cells (n=2). Final data were normalized to IgG (negative immunoprecipitation control). ECM: extracellular matrix. TCA: Tricarboxylic acid cycle. ETC: electron transport chain. TF: transcription factor. FC: fold change. Statistics: one sample t-test with reference value 1 (D, E, F, H). * p.value < 0.05; ** p.value < 0.01; *** p.value < 0.001. Error bars indicate s.e.m.

Supplementary Figure 4. A. Quantification of total spermidine and spermine levels in PC3 cells grown with the indicated CM. **B**. Representation of label-free LC/MS proteomics data of the CMs produced by PC3-TRIPZ-Pgc1a cells that were transduced with lentiviral vectors (SRM-clover and control-clover) to overexpress spermidine synthase. **C**. Effect of SRM-clover overexpression on the 2D-cell proliferation (crystal violet) of PC3-PGC1α expressing cells. Data represents crystal violet staining and quantification of cells grown for 6 days. **D**. Schematic representation of the experimental approach for conditioned media production by PC3-PGC1α expressing cells with and without SRM overexpression. **E.** Quantification of PC3-luc cells co-injected in nude mice together with PGC1α non-expressing and expressing PC3 cells (left panel) with or without overexpression of SRM (right panel). (n=5 mice per group; 2

injections per mice; 10 tumors in total per group). Dotted squares highlight the two time phases of the experiment: tumor formation (F) and tumor growth (G) **F**. Validation of SRM silencing in PC3 cells by RT-qPCR (n=3). **G**. Effect of SRM silencing on 2D-cell proliferation of PC3 cells (n=3). **H**. Schematic representation of the experimental approach for conditioned media production by PC3 cells with SRM silencing. D or Dox: doxycyline. Statistics: unpaired-t-test (A, B), one sample t-test with reference value 1 (D). * p.value < 0.05; ** p.value < 0.01; *** p.value < 0.001. CM: conditioned media. Ctrl: Control. D or Dox: doxycyline. Ns: not significant. Error bars indicate s.e.m.

Supplementary Figure 5. A. Analysis of SRM mRNA in PCa patients stratified according to the mean expression of a PGC1α-ERRα signature (1). **B**. Correlation analysis between PGC1α-ERRα signature mean expression and SRM mRNA expression in primary tumor specimens of different prostate cancer datasets. Sample sizes: Grasso, n=45; Taylor, n=131; Glinsky, n=79 and TCGA provisional, n=497. Statistics: Mann Whitney test (A) Spearman correlation Rho/ρ (B). * p.value < 0.05; ** p.value < 0.01; *** p.value < 0.001. Error bars indicate s.e.m. p.v: p-value.
