# Supplementary material for: Secreted spermidine synthase reveals a paracrine role for PGC1α-induced growth suppression in prostate cancer
Source: Cell Death Dis. 2025 Apr 23;16(1):330. doi: 10.1038/s41419-025-07639-4 (PMC12019391; doi:10.1038/s41419-025-07639-4)
Supplement: Supplementary file 10 — Uncropped WB [file 41419_2025_7639_MOESM10_ESM.pptx]

## Slide 1
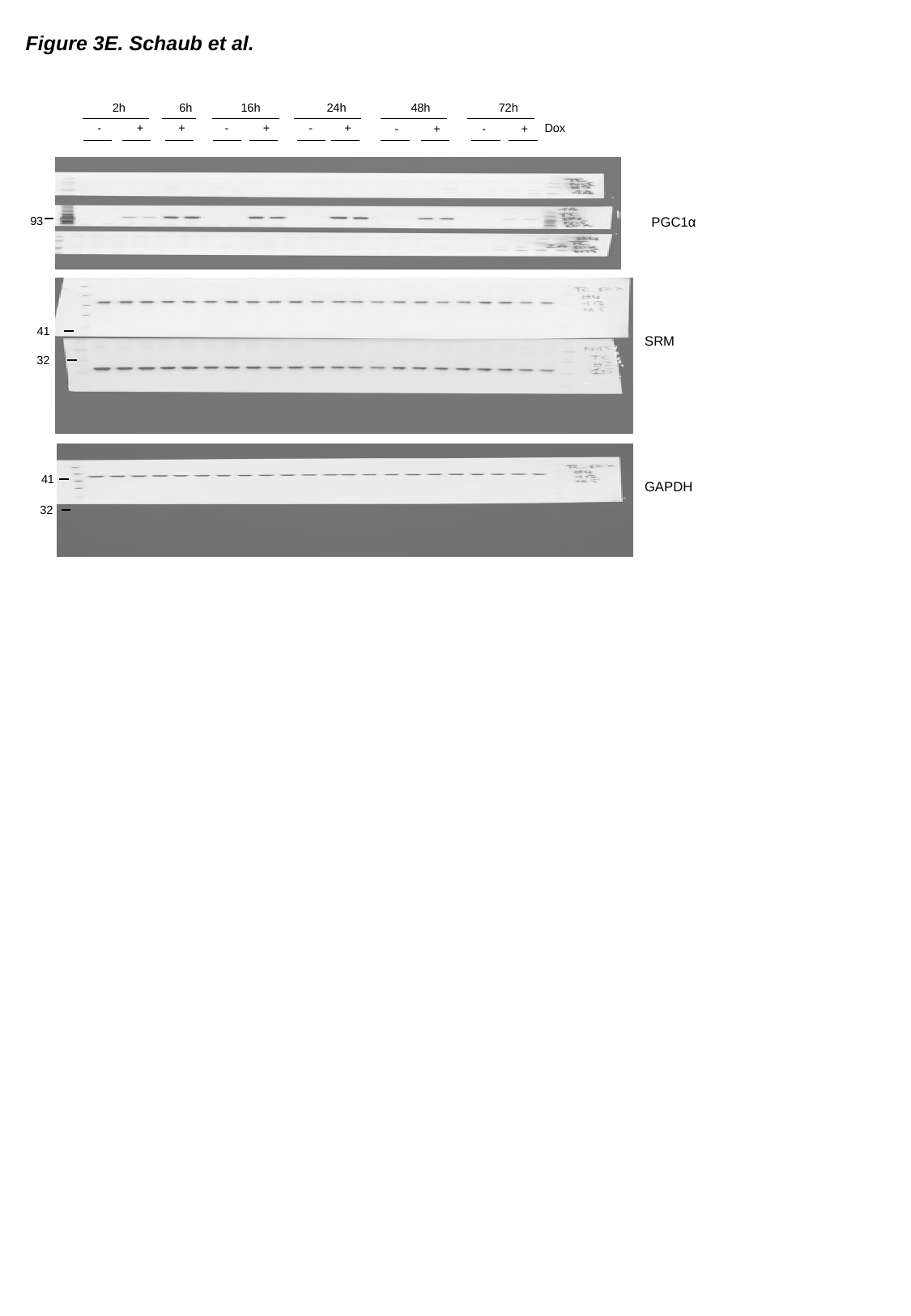

Figure 3E. Schaub et al.
 2h
 6h
16h
24h
48h
72h
-
+
-
+
Dox
+
-
+
-
+
-
+
93
PGC1α
41
SRM
32
41
GAPDH
32

## Slide 2
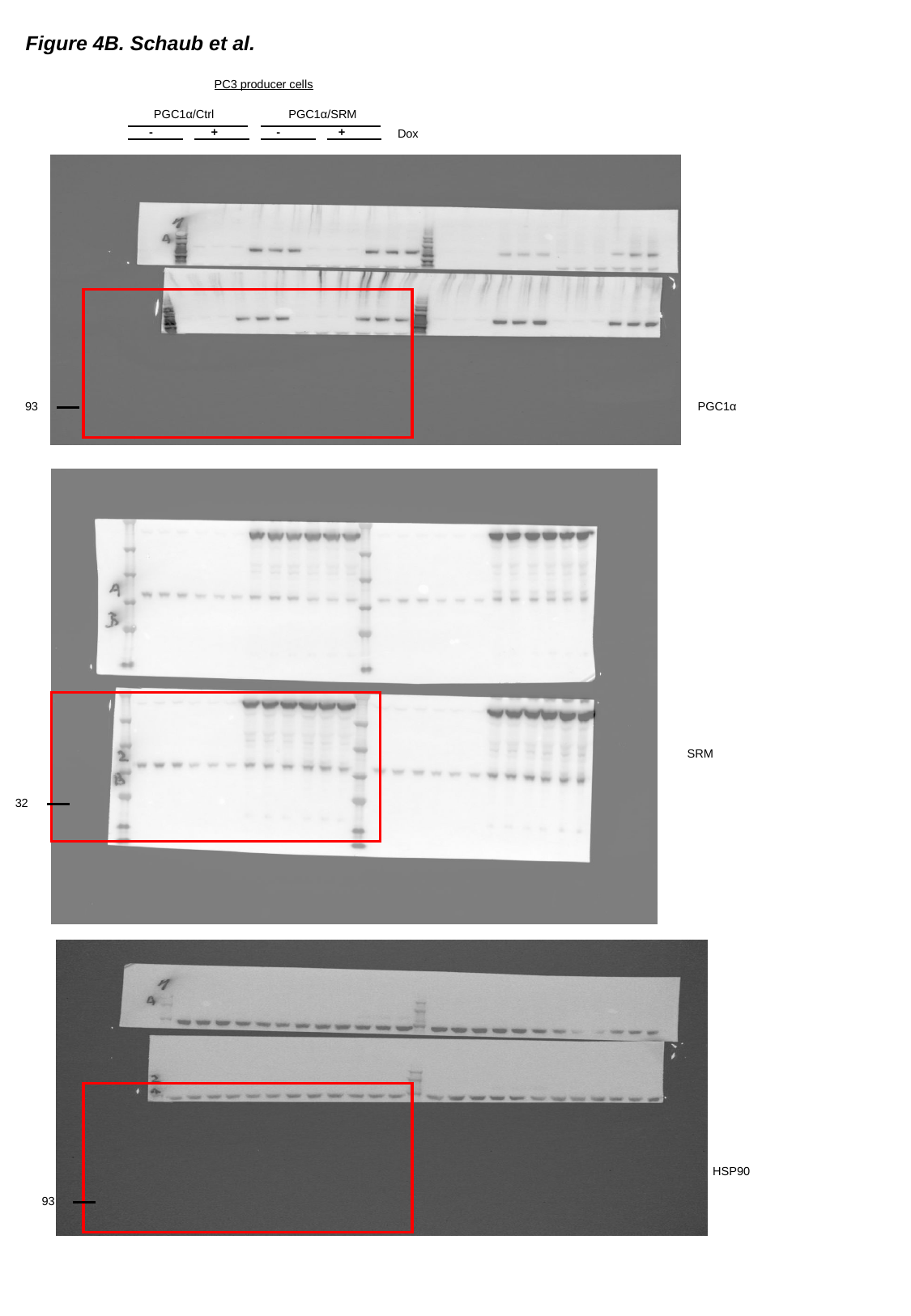

Figure 4B. Schaub et al.
PC3 producer cells
PGC1α/Ctrl
PGC1α/SRM
 - + - +
Dox
93
PGC1α
SRM
32
HSP90
93

## Slide 3
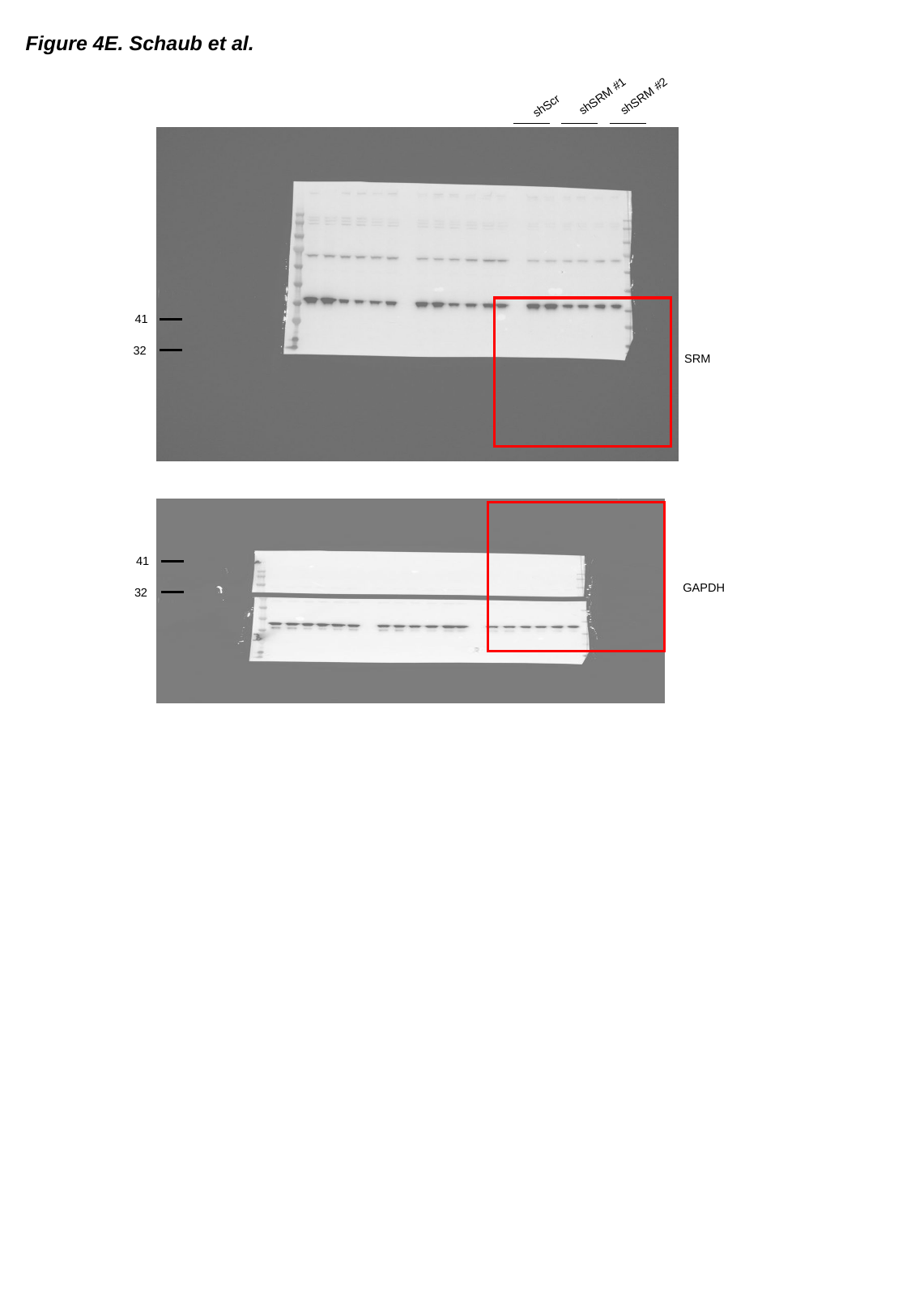

Figure 4E. Schaub et al.
shSRM #1
shSRM #2
shScr
41
32
SRM
41
GAPDH
32

## Slide 4
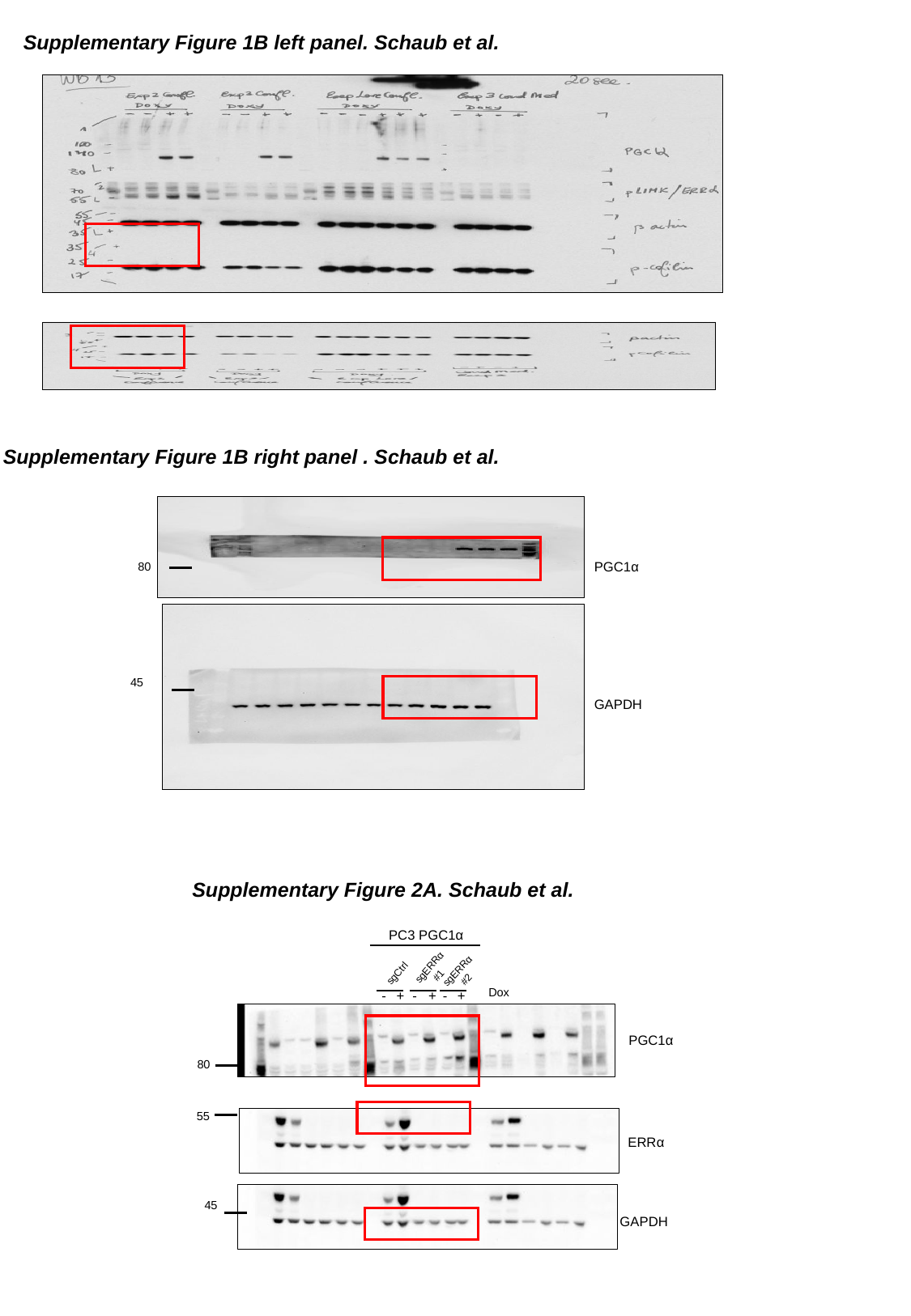

Supplementary Figure 1B left panel. Schaub et al.
140
45
Supplementary Figure 1B right panel . Schaub et al.
PGC1α
80
45
GAPDH
Supplementary Figure 2A. Schaub et al.
PC3 PGC1α
sgERRα
#1
sgERRα
#2
sgCtrl
Dox
-
+
-
+
-
+
PGC1α
80
55
ERRα
45
GAPDH
GAPDH

## Slide 5
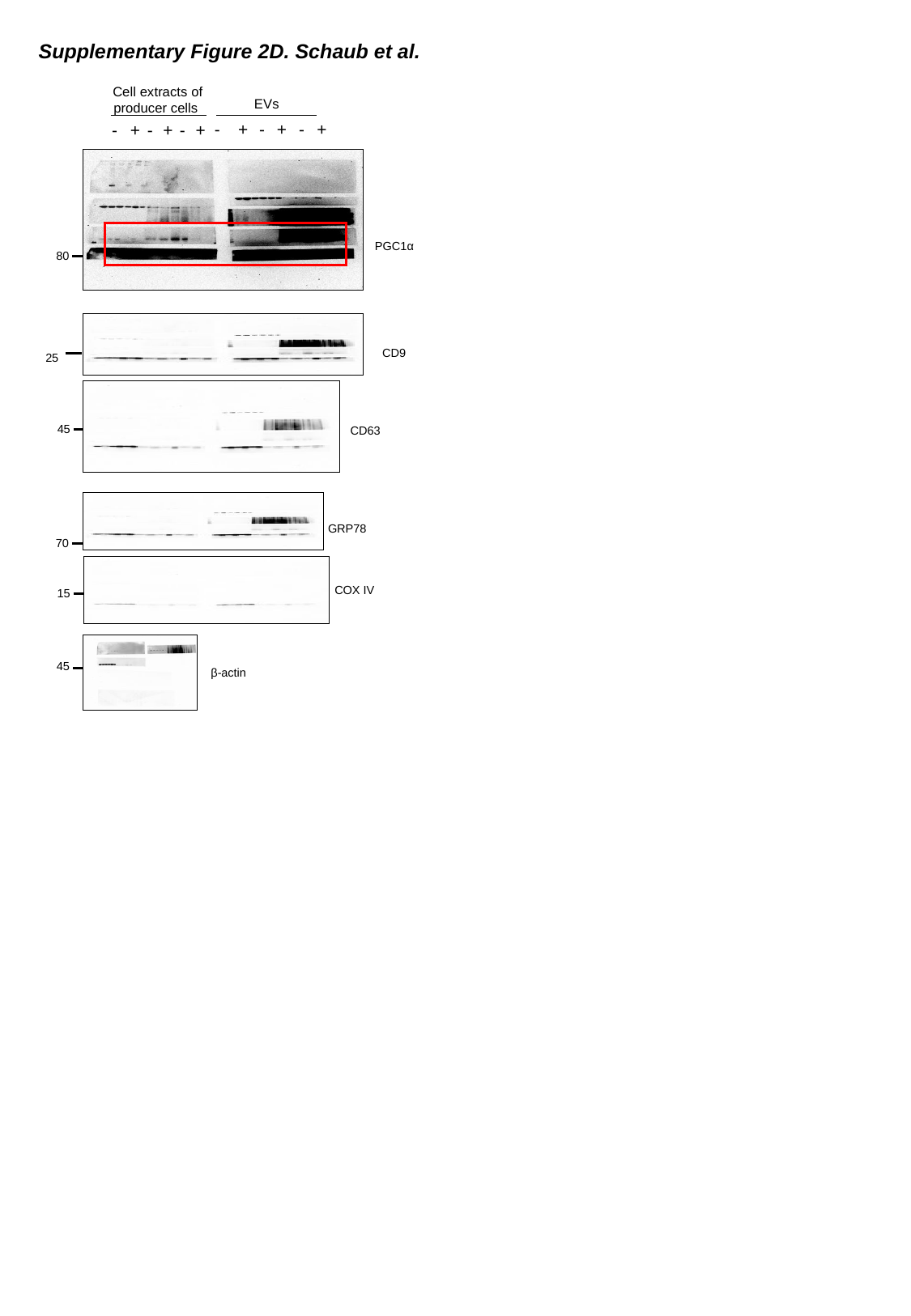

Supplementary Figure 2D. Schaub et al.
 Cell extracts of
producer cells
 EVs
-
+
-
+
-
+
-
+
-
+
-
+
PGC1α
80
CD9
25
45
CD63
GRP78
70
COX IV
15
45
β-actin

## Slide 6
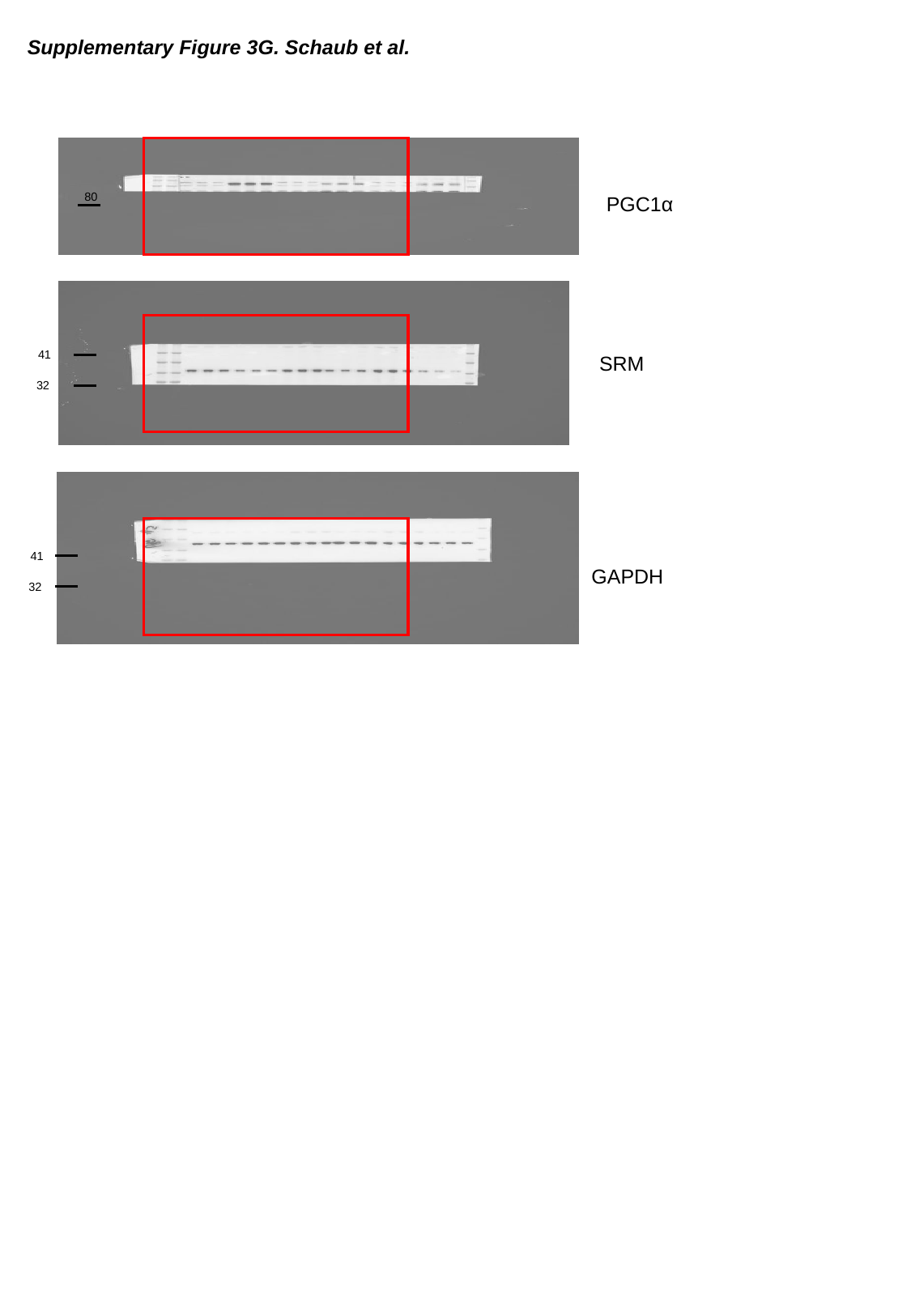

Supplementary Figure 3G. Schaub et al.
80
PGC1α
41
SRM
32
41
GAPDH
32
